# Utility of Visual Evoked Potentials (VEPs) study in the evaluation of visual pathway dysfunction in diabetics without retinopathy: correlations with diabetic peripheral neuropathy and other clinical findings

**DOI:** 10.22336/rjo.2024.22

**Published:** 2024

**Authors:** Kumar Ashok, Panjwani Ankit, Pandey K Nitin, Kumar Sanjeev

**Affiliations:** * Department of Neurology, Indira Gandhi Institute of Medical Sciences, Patna

**Keywords:** visual evoked potential (VEP), P-100 latency, diabetes mellitus, peripheral neuropathy

## Abstract

**Aim and objectives:** Visual dysfunction in diabetes mellitus (DM) is multifactorial and can be due to vascular disease, and metabolic abnormalities that can affect the retina, optic nerve, and visual pathways. Visual evoked potential (VEP) is an electrophysiological test that can quantify the functional integrity of the visual pathways from the retina via the optic nerves, and optic tracts to the visual cortices. In this study, we aimed to investigate the visual pathway dysfunction among diabetics without retinopathy compared with healthy controls and to look for any correlation with diabetic neuropathy, duration of diabetes, or HbA1c level.

**Methods:** The study included 75 diabetic patients and 75 age and sex-matched controls. VEPs were recorded using the pattern reversal stimulation method on the Medtronic EMG EP machine, and P100 latency and N75-P100 amplitude were recorded in both diabetic patients and healthy controls.

**Results:** Mean P100 latency was significantly prolonged and N75-P100 amplitude significantly reduced among diabetic cases compared to healthy controls (p < 0.001). Among diabetics with peripheral neuropathy, P100 latency was significantly prolonged and N75-P100 amplitude was significantly reduced compared to diabetics without peripheral neuropathy. A significant positive correlation of VEP P100 latency (p < 0.001) and a negative correlation with N75-P100 amplitude (p < 0.001) with duration of disease were also found.

**Conclusion:** VEP changes are observed in diabetics before the development of retinopathy or peripheral neuropathy indicating optic pathway dysfunction, which precedes the development of these complications. Early preclinical visual pathway dysfunction can warrant taking the necessary measures to reduce diabetic complications.

**Abbreviations:** DM = Diabetes Mellitus, VEP = Visual Evoked Potential, HbA1c = Hemoglobin A1 c, MRI = Magnetic Resonance Imaging, EEG = Electroencephalography, P100 = Positive wave peak at latency 100 ms (millisecond), N75 = Negative wave peak at latency 75 ms (millisecond), N145 = Negative wave peak at latency 145 ms (millisecond), OCT = Optical coherence tomography, PRVEP = Pattern Reversal Visual Evoked Potential, NCS = Nerve Conduction Study, SSR = Sympathetic Skin Response, IL1 = Interleukin-1, LIF = Leukemia inhibitory factor, CNTF = Ciliary neurotrophic factor, TNF alpha = Tumor necrosis factor-alpha, TGF-beta = Transforming growth factor-beta

## Introduction

Diabetes mellitus (DM) is a global epidemic and currently accounts for about 425 million cases worldwide, which are expected to reach 642 million by 2040. In 2017, there were 72 million cases of diabetes in India with a prevalence of 8.8, and are expected to reach 101.2 million by 2030 [**1**].

Peripheral and autonomic neuropathy are known complications of diabetes mellitus. Current research is looking for peripheral and central nervous system involvement among diabetics. To do so, among various electrophysiological tests available, VEP has been implicated as a simple and non-invasive test.

VEPs are electrical potential differences recorded from the scalp overlying visual cortex, which occur in response to visual stimuli [**2**]. VEPs are extracted from EEG by signal averaging. They are considered better than MRI in quantifying the functional integrity of the visual pathway [**3**]. Three waveforms are identified in VEP, of which N75 and N145 are negative and P100 is positive. P100 waveform has the greatest reproducibility among them. Neural generators of the waveforms of VEP are not clearly defined, but functional MRI and multichannel scalp recordings have shown that the visual cortex is responsible for the N75 wave, the dorsal extrastriate cortex for P100, and several areas including the deep parietal lobe is responsible for N145 [**3**].

Abnormalities in VEP arise before diabetic retinopathy and neuropathy signs become clinically detectable and, hence, anomalies that precede clinically evident structural alterations in the retina and visual pathways, can be detected by this objective and non-invasive electrophysiological technique [**4**].

Optical coherence tomography (OCT) is another non-invasive test proven to be useful in measuring retinal thickness. While it is useful in detecting changes in diabetic retinopathy, its utility in diabetics before the development of retinopathy is doubtful [**5**,**6**]. OCT primarily assesses the retinal pathologies and is less sensitive than VEP in patients with neuromyelitis optica, multiple sclerosis, and optic neuritis [**7**-**9**]. Thus, VEP can detect retinal and optic nerve pathologies with a higher sensitivity than OCT. 

Hence, the present study was conducted to search the subclinical detection of visual pathway involvement before the development of retinopathy in diabetes by visual evoked potentials (VEP) test and to assess the utility of this test in early prediction of development of diabetic retinopathy and diabetic peripheral neuropathy.

## Aims and objectives

1. To compare VEP in patients with diabetes mellitus to those of healthy controls. 

2. To study the association of VEP changes with diabetic peripheral neuropathy, duration of disease, and HbA1c level.

## Materials and methods

It was a case-control study with 1:1 age and sex matching. The study was carried out among the OPD and indoor patients of diabetes mellitus under the Neurology Department at a tertiary care institute in Eastern India from July 2021 to December 2022.

*Minimum sample size*

The minimum sample size was calculated using Dupont’s method [**10**,**11**], with the following considerations:

α= 5%

Power of study = 80%

r = 0.2

The proportion of diabetics without retinopathy having abnormal VEP (based on previous studies) [**12**] = 40%

The proportion of controls having abnormal VEP (based on previous studies) [**12**] = 13.75%

Odd’s ratio based on previous studies = 4.1

Minimal sample size for both cases and controls = 53

*Inclusion criteria*

1. Patients with diabetes mellitus.

2. Patients aged 18 years or more.

3. All patients who gave an informed written consent.

*Exclusion criteria*

1. Patients or their guardians, who did not give their written informed consent.

2. Patients below 18 years of age.

3. Patients with any stage of diabetic retinopathy. 

4. Patients with neurodegenerative disorders, a history of stroke, and demyelinating disorders.

5. Patients with ocular disorders.

6. Patients taking neurotoxic drugs.

7. Patients with visual acuity less than 6/18.

*Procedure*

The study was conducted on 150 subjects, 75 cases, and 75 controls. The cases with diabetes mellitus were selected from the patients attending the Neurology and Endocrinology OPD at our tertiary care center during 2021-2022. Age and sex-matched controls (1:1) were selected from the general population. The ethical committee clearance (No. 283/IEC/IGIMS/2021) and informed consent of the subjects were obtained.

Direct and indirect ophthalmoscopy were done and subjects with any stage of diabetic retinopathy or any ocular disorders were excluded from the study. Visual acuity was tested using Snellen’s chart and subjects with visual acuity < 6/18 were excluded from the study. Personal details including name, age, sex, and contact details were recorded. A detailed clinical history was noted and a thorough physical examination was performed. Routine blood investigations including complete blood counts, renal and liver function tests, and HbA1c were performed. 

**Fig. 1:**
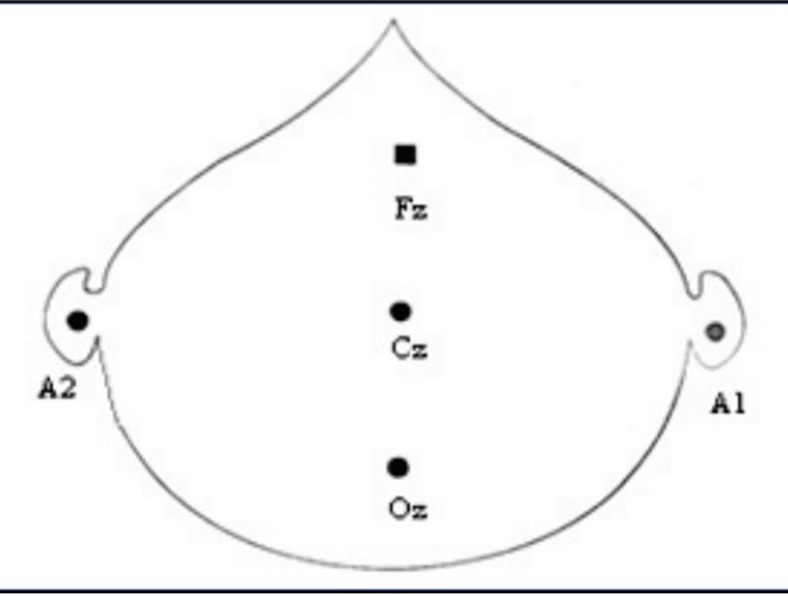
Position of recording electrodes in VEP (Oz - active, Fz - reference electrode, Cz - ground electrode)

**Fig. 2:**
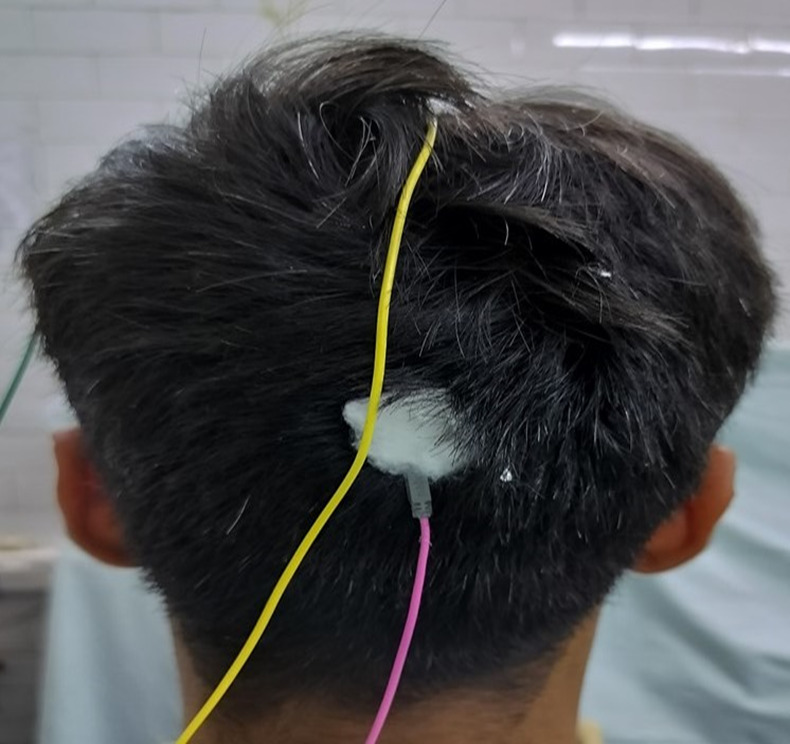
Illustrated image showing the position of electrodes in a study subject

VEPs were recorded with a PC-based, 2-channel, Medtronic EMG EP machine and standard silver cup electrodes. A VEP monitor displaying the pattern reversal stimulus on a checkerboard was used. A montage consisting of one channel was used for the VEP recording. The distance between the screen showing the checkerboard pattern and the subject was 100 cm. The electrode placement site was cleaned and electrodes were placed with an electrode paste. To ensure the reproducibility of electrode placement, they were placed according to the International 10/20 system relative to bony landmarks proportionally to the size of the head. The anterior/posterior midline measurements were based on the distance between the nasion and the inion over the vertex. The active electrode was placed in the middle of the variation zone of the calcarine fissure at Oz, which is the highest point on the occiput. The reference electrode was placed at Fz or 12 cm above the inion. The ground electrode was placed over the vertex or the forehead at Cz. [**13**] (**Fig. 1**,**2**).

**Fig. 3:**
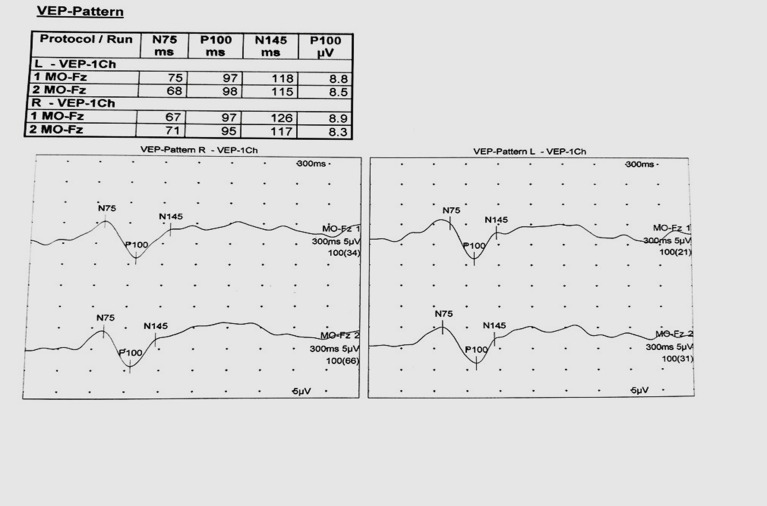
VEP recording of a normal subject showing N75, P100 and N145 peaks latency (ms) with P100 amplitude (µv)

VEPs were performed with the patients wearing the best refractive correction. Subjects were explained to fix their gaze at the center of the checkerboard, a red dot, to avoid interference in potentials due to the movement of the eyeball. Monocular stimulation was applied. Pattern stimuli were presented with a black and white (12 X 16) checkerboard reversing alternately at 2 Hz, contrast 99%, and a full-field display. Responses to 200 stimuli were recorded. The signals were amplified and displayed as a waveform. The amplifier band-pass filters were set at 1-30 Hz. The waveform consists of components of opposite polarity, negative ones are denoted as N, and positive ones are denoted as P. The usual pattern reversal VEP (PRVEP) waveform consists of an initial negative peak (N75), followed by a large positive peak (P100), and followed by another negative peak (N145) (**Fig. 3**) [**13**].

The parameters recorded were: the latencies of the waves N75, P100, and N145 (in milliseconds) and the peak-to-peak amplitudes of the waves N75-P100 (in microvolts).

Nerve conduction studies were done in all the study subjects. Five standard NCS (motor and sensory NCS of the median nerve, ulnar nerve, motor NCS of the peroneal nerve, and sensory NCS of the sural nerve) were determined in all the subjects according to Kimura’s methods and criteria of interpretation using Nicolet EMG EP II equipment [**14**]. 

Sympathetic skin response (SSR) testing was done to assess for small fiber neuropathy in all study subjects. A surface electrode was placed on the palm and sole and a reference electrode was placed on the dorsal surface of the same limb. The sweep was set to 1 second per division, gain at 100 microvolts per division, and band pass at 2 kHz. An electrical stimulus of 0.1 ms in duration and 10-20 mA in intensity was used and applied to the ipsilateral wrist and ankle. 

Various NCS and VEP parameters were compared between the cases and controls. Cases were further divided into two groups based on the presence of diabetic neuropathy electrophysiologically. Various NCS and VEP parameters were compared between the diabetic patients without peripheral neuropathy and those with peripheral neuropathy.

*Case definitions*

• Diabetes mellitus was defined as either a history of diagnosis of diabetes mellitus or receiving treatment in the form of oral hypoglycemic drugs or insulin or fasting plasma glucose ≥ 126 mg/dL or postprandial plasma glucose ≥ 200 mg/dL or HbA1c > 6.5% in previously undiagnosed patients according to 2021 criteria of American Diabetes Association [**15**].

• Diabetic neuropathy: an abnormality (≥ 99th or ≤ 1st percentile) of any attribute of nerve conduction in two separate nerves (among sural sensory, ulnar sensory, and median sensory nerves, and peroneal, tibial, median, and ulnar motor nerves), one of which must be the sural nerve [**16**].

## Results

Various baseline characteristics among the study subjects (**Table 1**).

**Table 1:** Various characteristics among the study subjects

Variable	Cases (n)
Mean age	56.12 ± 9.71 years
Males	64% (48)
Females	36% (27)
Peripheral neuropathy	34.67% (26)
Mean HbA1c	8.29 ± 1.72%
The mean duration of diabetes	5.47 ± 2.75 years

Mean P100 latency and N75-P100 amplitude in the right eye and left eye among cases were comparable. Similarly, mean P100 latency and N75-P100 amplitude in the right eye and left eye among controls were comparable (**Table 2**). Hence, hereafter, P100 latency and N75-P100 amplitude of the right eye were considered for further analyses.

**Table 2:** Comparing mean P100 latency and N75-P100 amplitude of the right and left eye in the study groups (paired t-test)

VEP P100 latency (ms)		Mean ± SD (ms)		P-value
		Right eye	Left eye	
Group	Cases (75)	111.05 ± 11.2	110.66 ± 16.37	0.909
	Controls (75)	99.23 ± 3.52	99.62 ± 3.52	0.094
VEP N75-P100 amp (µv)		Right eye	Left eye	P-value
Group	Cases (75)	4.73 ± 2.01	4.66 ± 1.76	0.562
	Controls (75)	5.37 ± 1.22	5.21 ± 1.26	0.228

VEP P100 latency and N75-P100 amplitude were compared among the study subjects using paired t-tests (**Table 3**). P100 latency was significantly prolonged among cases as compared to controls. N75-P100 amplitude was significantly reduced in the cases.

**Table 3:** Comparison of various VEP parameters among study subjects (paired t-test)

Group (n)	Mean VEP P100 latency (ms)	P value	Mean VEP N75-P100 amplitude (µV)	P value
Cases (75)	111.05 ± 11.2	< 0.001	4.73 ± 2.01	0.012
Controls (75)	99.23 ± 3.52		5.37 ± 1.22	

Cases were further divided into those with peripheral neuropathy and those without peripheral neuropathy. A comparison of VEP parameters was done among them using an unpaired t-test (**Table 4**). P100 latency was significantly prolonged in cases with peripheral neuropathy and N75-P100 amplitude was significantly reduced compared to cases without peripheral neuropathy.

**Table 4:** Comparison of VEP parameters among cases with and without peripheral neuropathy (unpaired t-test)

Group (n)	Mean VEP P100 latency (ms)	P value	Mean VEP N75-P100 amplitude (µV)	P value
Diabetics with peripheral neuropathy (26)	119.40 ± 13.70	< 0.001	3.59 ± 1.59	< 0.001
Diabetics without peripheral neuropathy (49)	106.23 ± 5.02		5.35 ± 1.92	

A comparison of VEP parameters of diabetics with and without peripheral neuropathy and controls was done using an unpaired t-test (**Table 5**).

**Table 5:** Comparison of VEP parameters of diabetics with and without peripheral neuropathy and controls (unpaired t-test)

Group (n)	Mean VEP P100 latency (ms)	P value	Mean VEP N75-P100 amplitude (µV)	P value
Diabetics without peripheral neuropathy (49)	106.23 ± 5.02	< 0.001	5.35 ± 1.92	0.92
Controls (75)	99.22 ± 3.52		5.37 ± 1.22	
Diabetics with peripheral neuropathy (26)	119.40 ± 13.70	< 0.001	3.59 ± 1.59	< 0.001
Controls (75)	99.22 ± 3.52		5.37 ± 1.22	

A comparison of VEP parameters among diabetics with and without recordable sympathetic skin response (SSR) using an unpaired t-test was made (**Table 6**).

**Table 6:** Comparison of VEP parameters among diabetics with and without recordable sympathetic skin response (SSR) (unpaired t-test)

Group (n)	Mean VEP P100 latency (ms)	P value	Mean VEP N75-P100 amplitude (µV)	P value
Diabetics with present SSR (27)	106.68 ± 3.96	0.013	5.65 ± 1.64	0.002
Diabetics with absent SSR (48)	113.12 ± 12.82		4.22 ± 2.00	

VEP P100 latency (Spearman’s Correlation coefficient, r = 0.063, P-value = 0.592) and N75-P100 amplitude (Spearman’s Correlation coefficient, rs = 0.064, P-value = 0.584) did not show any correlation with HbA1c levels.

**Fig. 4:**
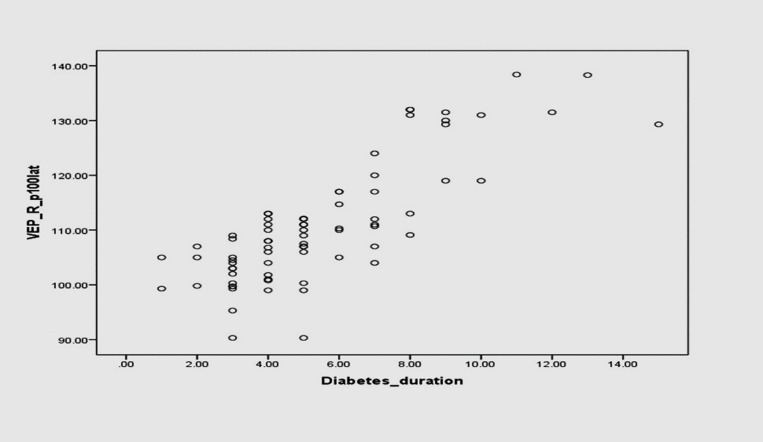
Scatter plot showing the correlation of VEP P100 latency with the duration of diabetes (Pearson’s correlation)

However, P100 latency showed a significant positive correlation with the duration of diabetes (Pearson’s Correlation coefficient, r = 0.811, **P-value < 0.001**) (**Fig. 4**).

**Fig. 5:**
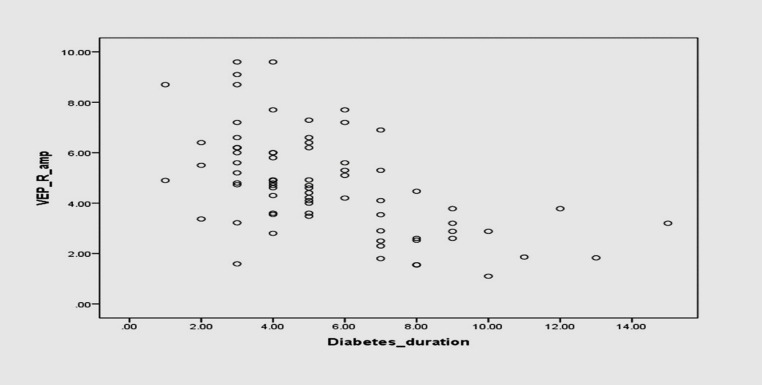
Scatter plot showing the correlation of VEP N75-P100 amplitude with the duration of diabetes (Pearson’s correlation)

N75-P100 amplitude showed a significant negative correlation (Pearson’s Correlation coefficient, r = -0.550, **P-value < 0.001**) with the duration of diabetes (**Fig. 5**).

## Discussion

The VEP indicates the function of the entire visual pathway from the retina to the visual cortex and primarily reflects the central retinal projection to the occipital poles. Thus, it is a sensitive tool to study the effects of diabetes mellitus on the visual system. In this study, we aimed to see the affection of the visual function before the development of retinopathy.

The mean age of cases in the present study was 56.12 ± 9.71. A similar age distribution has also been seen in the previous studies by Heravian et al., Khatoon et al., and Saadoun et al. [**12**,**17**,**18**].

Mean P100 latency in cases and controls were compared and P100 latencies among cases were significantly prolonged (p < 0.001), thus, implying that diabetic patients had significantly prolonged P100 latencies on VEP before the development of clinically evident retinopathy. Previous studies by Heravian et al., Khatoon et al., Gowri et al., Deak et al., Balakrishnan et al., and Saadoun et al. also had similar findings [**12**,**17**-**21**]. Due to functional disturbance in the visual conduction pathway rather than demyelination or axonal loss, it is also possible that early diabetic pre-retinopathy due to retinal ganglion cell loss may also contribute to P100 prolongation [**22**].

Mean N75-P100 amplitudes among cases and controls were compared. The difference between the cases and controls was statistically significant (p-value 0.012). Studies by Heravian et al. [**12**], Deak et al. [**20**], and Gupta et al. [**23**] also found the difference in N75-P100 amplitude to be significant. However, Khatoon et al. [**17**] and Saadoun et al. [**18**] found no statistically significant difference in N75-P100 amplitude among diabetic cases and controls. Thus, it implies an underlying axonal loss in the optic nerve and its pathway.

P100 latency of diabetic cases without peripheral neuropathy was significantly prolonged compared to controls (p-value < 0.001), indicating that P100 latency among diabetics is affected before the development of retinopathy and peripheral neuropathy. This was consistent with the findings by Gupta et al. [**23**]. However, Deak et al. [**20**] did not find a significant difference in the P100 latency of diabetics without peripheral neuropathy and controls. There was no significant difference in the N75-P100 amplitude among cases without peripheral neuropathy compared to controls. Gupta et al. reported a significant difference in amplitude in diabetics not having peripheral neuropathy compared to controls [**23**]. This difference could be attributed to the different criteria used for diabetic peripheral neuropathy in other studies. 

Diabetic patients were divided into two groups based on peripheral neuropathy and VEP parameters were compared among them. The cases with peripheral neuropathy had significantly prolonged P100 latency and reduced N75-P100 amplitude as compared to the cases that did not have peripheral neuropathy. Reduction in N75-P100 amplitude with the development of peripheral neuropathy in the present study could indicate initiation of axonal loss. Balakrishnan et al. compared P100 latency among diabetics with and without peripheral neuropathy and found no significant difference [**21**]. This could be due to different criteria used to define diabetic peripheral neuropathy.

No significant correlation of P100 latency (p = 0.592) or N75-P100 amplitude (p = 0.584) was found with HbA1c levels. Heravian et al., Balakrishnan et al., Saadoun et al., and Dolu et al. also reported no significant correlation between P100 latency and HbA1c levels [**12**,**18**,**21**,**24**]. Saadoun et al. also reported no correlation of N75-P100 amplitude with HbA1c levels [**18**].

In this study, P100 latency was significantly prolonged in patients with absent sympathetic skin response (SSR) test compared to normal SSR. N75-P100 amplitude was also significantly reduced among absent SSR patients. These results indicated abnormal VEP parameters even if patients had small fiber neuropathy or sudomotor dysfunction.

In the present study, the duration of diabetes mellitus showed a significant correlation with P100 latency (p-value < 0.001) and N75-P100 amplitude (p-value < 0.001). Khatoon et al. [**17**], Balakrishnan et al. [**21**], and Dolu et al. [**24**] also found that P100 latency prolongation correlated well with the duration of diabetes mellitus. But, Heravian et al. [**12**] and Saadoun et al. [**18**] found no correlation between the duration of DM and P100 latency prolongation. This may be due to reduced velocity of nerve conduction in the optic nerve, whereas in shorter disease duration inner retinal layers suffer neurosensory deficits but photoreceptors remain unaffected [**25**].

The exact pathophysiology for visual pathway involvement is still unknown. It may be multifactorial, similar to peripheral neuropathy, with both metabolic and vascular factors playing a role. Ischemia, reduced protein synthesis, depleted myoinositol, and high sorbitol levels have been shown to result in nerve fiber loss in the peripheral nerves. Hence, possibly even the optic nerve fibers may suffer from these diabetes-induced changes. Neuropoietic cytokines including interleukin-1 (IL-1), IL-6, leukemia inhibitory factor (LIF), ciliary neurotrophic factor (CNTF), tumor necrosis factor-alpha (TNF-alpha), and transforming growth factor-beta (TGF-beta), exhibit pleiotropic effects on the homeostasis of the glia and the neurons in the central, peripheral, and the autonomic nervous systems. These cytokines are produced locally by macrophages, lymphocytes, mast cells, fibroblasts, and sensory neurons [**21**]. The accumulation of these mediators probably delays the conduction in the visual pathway, which can be the probable cause of the delay in the latencies found in diabetics as compared to the healthy controls. With the increase in the duration of diabetes, the accumulation of these mediators also increases, which can cause further delay in the latencies in diabetics with more duration of the disease [**26**].

## Conclusion

P100 latency is significantly prolonged among diabetics without peripheral neuropathy as compared to healthy individuals indicating that retinal ganglion cell damage precedes the development of diabetic retinopathy and peripheral neuropathy. P100 latency is also significantly prolonged and N75-P100 amplitude is significantly reduced in diabetics with peripheral neuropathy as compared to those without peripheral neuropathy or healthy controls indicating that the development of peripheral neuropathy among diabetics leads to further damage to the optic pathway and prolongation of latency along with reduction of amplitude, which could be a potential predictor of development of peripheral neuropathy among diabetics. 

Visual pathway involvement in diabetes mellitus could be multifactorial, not necessarily due to retinopathy, and VEP can help in the early and subclinical detection of visual pathway involvement before the development of retinopathy. Prolongation of P100 latency and reduction in N75-P100 amplitude in patients with diabetic peripheral neuropathy indicates that involvement of visual pathway in VEP can be a predictor of diabetic neuropathy, but further studies are needed with serial monitoring of VEP and nerve conduction studies for a better demonstration of this correlation. The prolongation of P100 latency and reduced N75-P100 amplitude can be a poorer visual outcome predictor in such patients.

**Conflict of Interest Statement**

The authors state no conflict of interest.

**Informed Consent and Human and Animal Rights Statement**

Informed consent has been obtained from all individuals included in this study.

**Authorization for the use of human subjects**

Ethical approval: The research related to human use complies with all the relevant national regulations, and institutional policies, as per the tenets of the Helsinki Declaration, and has been approved by the ethics committee of Indira Gandhi Institute of Medical Sciences, Patna, India (No. 283/IEC/IGIMS/2021).

**Acknowledgment**

We would like to thank our patients and working colleagues for their continuous support in this study.

**Sources of Funding**

None.

**Disclosures**

None. 
